# Supplementary material for: COCO/DAND5 inhibits developmental and pathological ocular angiogenesis
Source: EMBO Mol Med. 2021 Feb 15;13(3):e12005. doi: 10.15252/emmm.202012005 (PMC7933934; doi:10.15252/emmm.202012005)
Supplement: Supplementary file 1 — Expanded View Figures PDF [file EMMM-13-e12005-s001.pdf]

Expanded View Figures

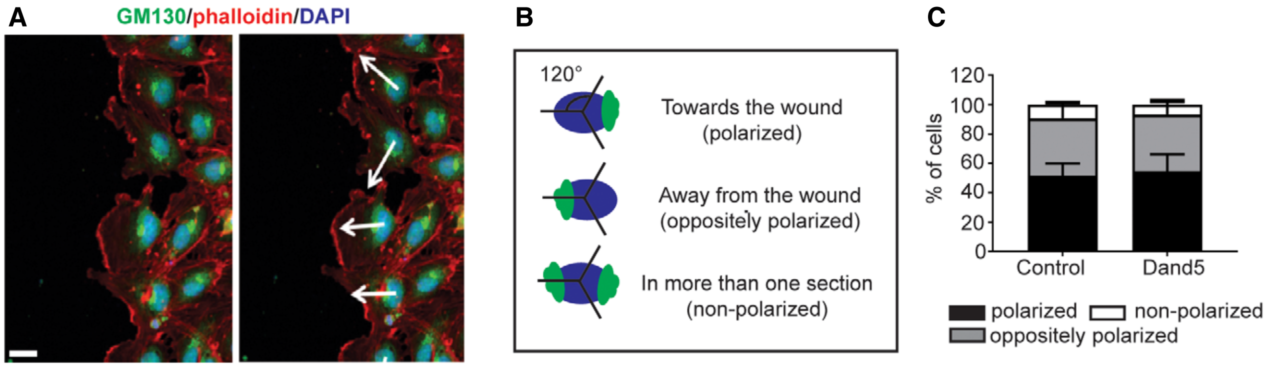

Figure EV1. COCO treatment does not affect front-rear polarity.

A Golgi (GM130) orientation of COCO-treated HUVECs. Scale bar, 20 µm.
B Schematic of the polarization quantification strategy.
C Quantification of cell polarity in COCO-treated HUVECs (n = 3; 100 cells/experiment).

Figure EV2. Effect of COCO on retinal proliferation and apoptosis.

A Schematic of the experimental strategy to assess proliferation and apoptosis of the retinal vasculature.
B, C Pictures and quantification of retinas injected 24 h prior with recombinant COCO, stained with IsoB4 and phospho-HistoneH3 antibody. ***P = 0.0007; (n = 11).
D, E Pictures and quantification of retinas injected 24 h prior with recombinant COCO, stained with IsoB4 and cleaved caspase 3 antibody; (n = 3–5 mice/group). Scale bars, 100 µm. Results are presented as mean ± SEM and statistical significance was analyzed by Mann–Whitney test. ns: not significant.

© 2021 The Authors

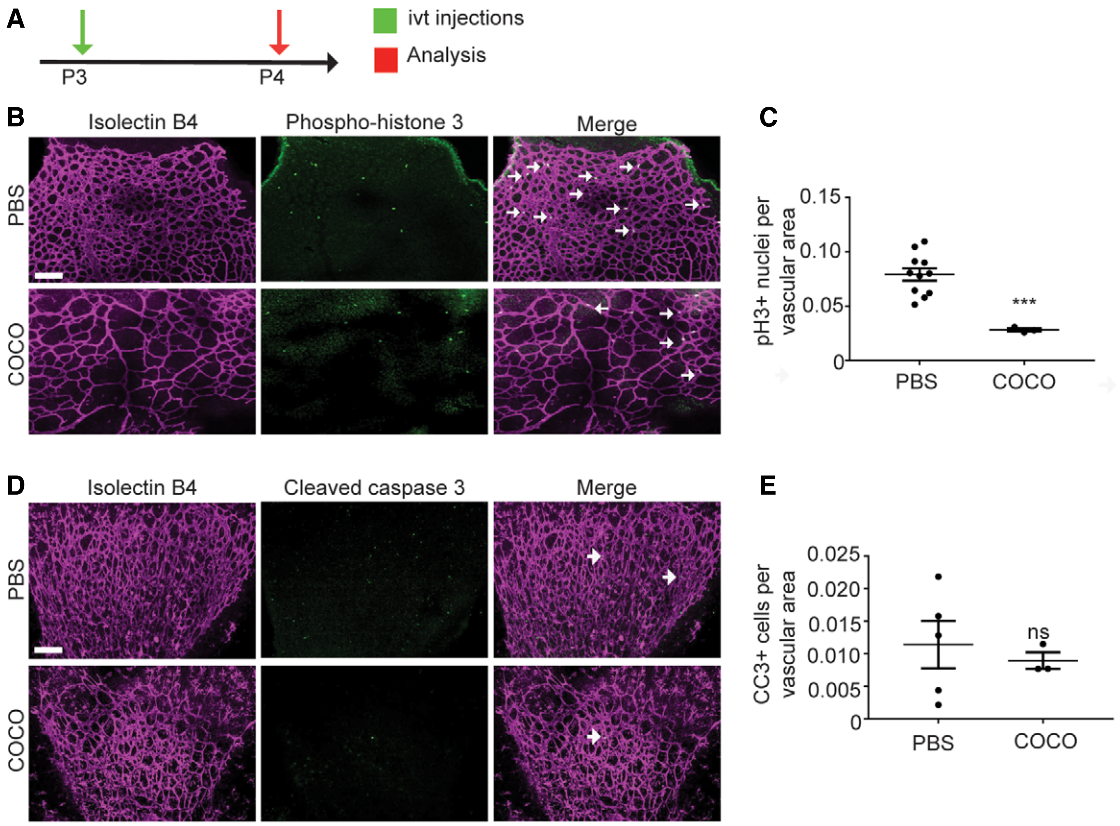

Figure EV2.

Figure EV3. Effect of the combination of COCO and VEGF inhibition on angiogenesis.

A Representative images of HUVECs sprouting in a fibrin gel with or without Flt1Fc (100 ng/ml) in the presence or absence of COCO. Scale bar, 75 μm.

B Quantification of the number of junctions/field of images shown in (A). **P* = 0.0378 (Untreated vs. Flt1Fc); ***P* = 0.0069 (COCO50 ng/ml vs. COCO50 ng/ml + Flt1Fc); (*n* = 3; 5 pictures/experiment).

C Retinal flat mounts of P5 mice injected with PBS, COCO, or Flt1Fc alone or in combination are stained with IB4 (negative images of the fluorescent signal). Scale bar, 100 μm.

D Quantification of number of branchpoints. Results are presented as mean ± SEM and statistical significance was analyzed by two-way ANOVA. **P* = 0.0151 (PBS vs. Flt1Fc); ***P* = 0.0060 (PBS vs. COCO); ***P* = 0.0079 (PBS vs. COCO + Flt1Fc); n.s. = non significant; (*n* = 7).

© 2021 The Authors

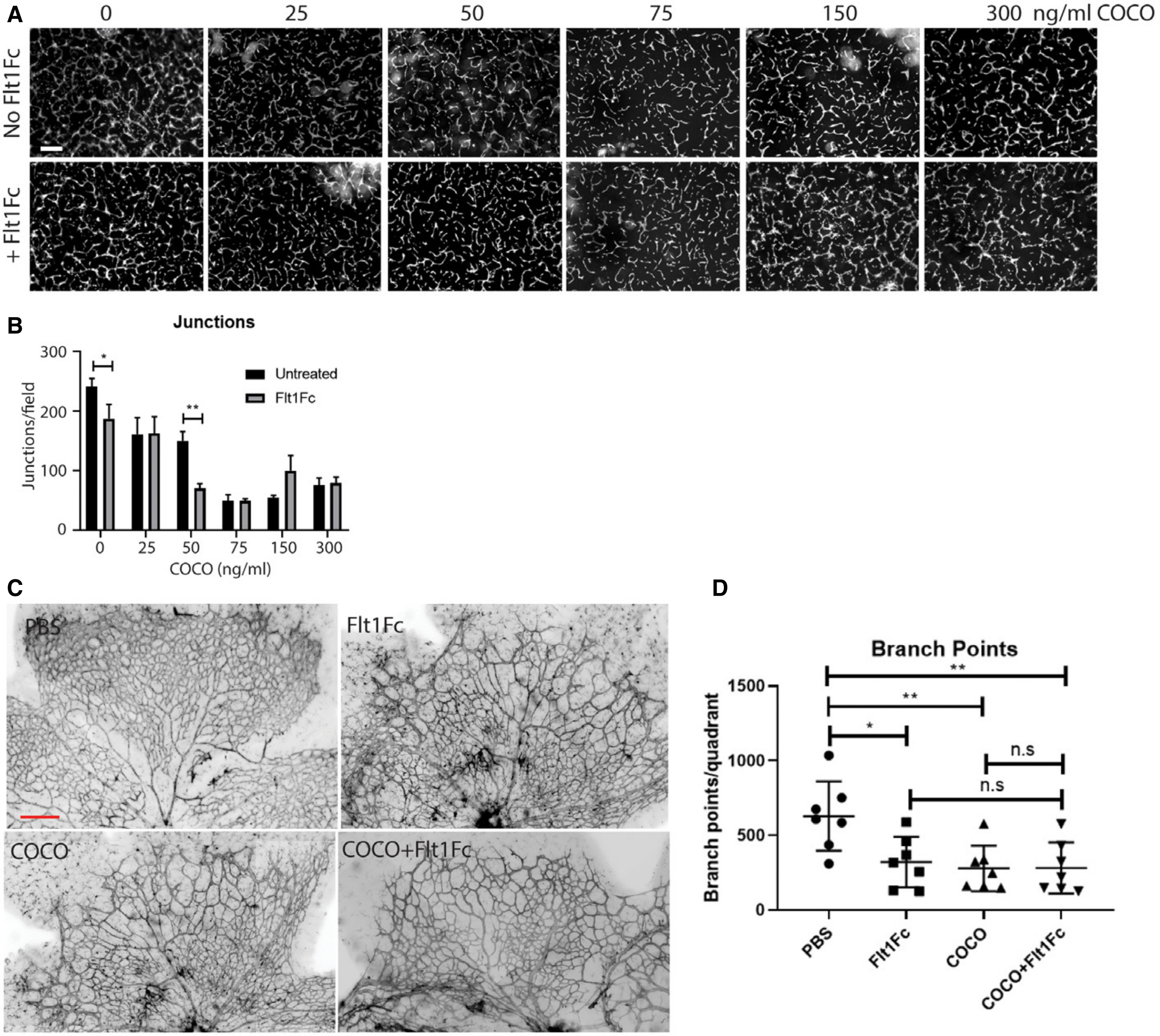
A
0
25
50
75
150
300 ng/ml COCO
No Flt1Fc
+ Flt1Fc
B
Junctions
Junctions/field
Untreated
Flt1Fc
COCO (ng/ml)
C
PBS
Flt1Fc
COCO
COCO+Flt1Fc
D
Branch Points
Branch points/quadrant
n.s
PBS
Flt1Fc
COCO
COCO+Flt1Fc

Figure EV3.

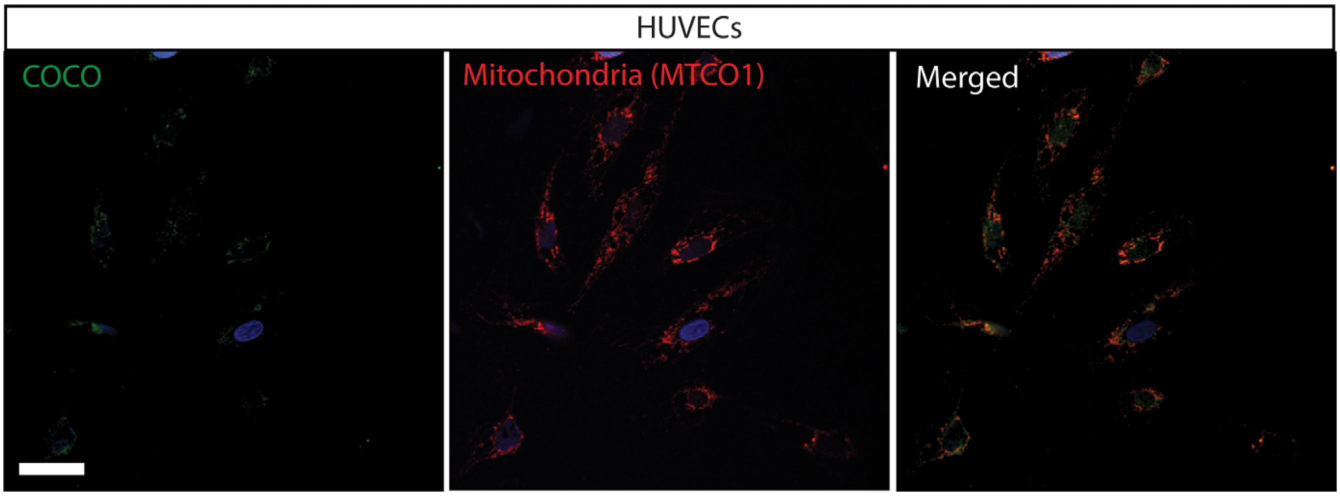

Figure EV4. COCO immunostaining in unstimulated HUVECs.

COCO and MTCO1 immunofluoresence staining of unstimulated HUVECs. Scale bar 25 μm.

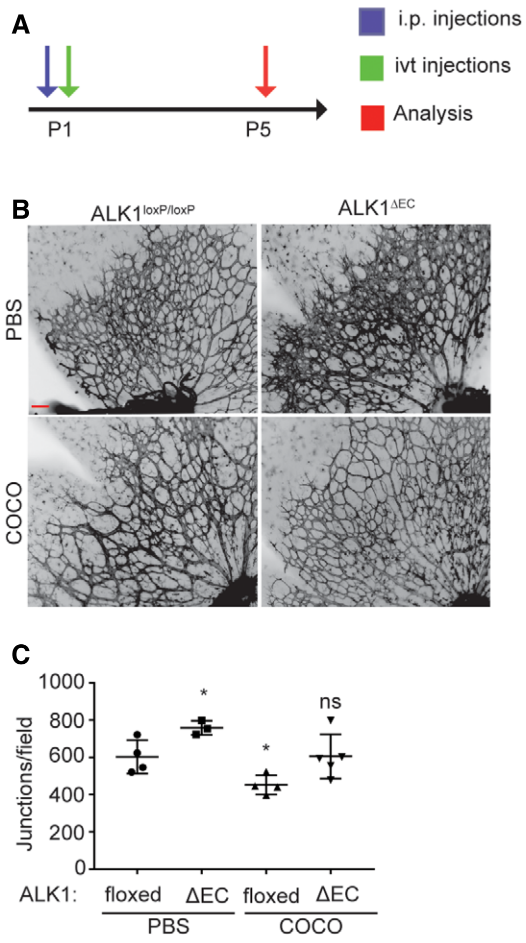

Figure EV5. Effect of COCO delivery on retinal development in control ($Alk1^{loxP/loxP}$) or Alk1ΔEC mice.

- A Schematic of the experimental strategy to assess early formation of the retinal vasculature in tamoxifen-inducible Alk1ΔEC mice.
- B Retinal flat mounts of P5 mice. Scale bar, 100 μm.
- C Quantification of number of branchpoints. *N* = 4 animals/group. Results are presented as mean ± SEM and statistical significance was analyzed by Mann–Whitney test. **P* = 0.0126 for ΔEC(PBS); **P* = 0.0152 for floxed (COCO); ns: not significant; (*n* = 3–5/group).

 © 2021 The Authors

WILEY

Author Query Form

Journal: EMMM

Article: 202012005

Dear Author,

During the copyediting of your manuscript the following queries arose.

Please refer to the query reference callout numbers in the page proofs and respond to each by marking the necessary comments using the PDF annotation tools.

Please remember illegible or unclear comments and corrections may delay publication.

Many thanks for your assistance.

AUTHOR: Please note that missing content in references have been updated where we have been able to match the missing elements without ambiguity against a standard citation database, to meet the reference style requirements of the journal. It is your responsibility to check and ensure that all listed references are complete and accurate.

Query reference	Query	Remarks
