# Supplementary figures and images for: COCO/DAND5 inhibits developmental and pathological ocular angiogenesis
Source: EMBO Mol Med. 2021 Feb 15;13(3):e12005. doi: 10.15252/emmm.202012005 (PMC7933934; doi:10.15252/emmm.202012005)

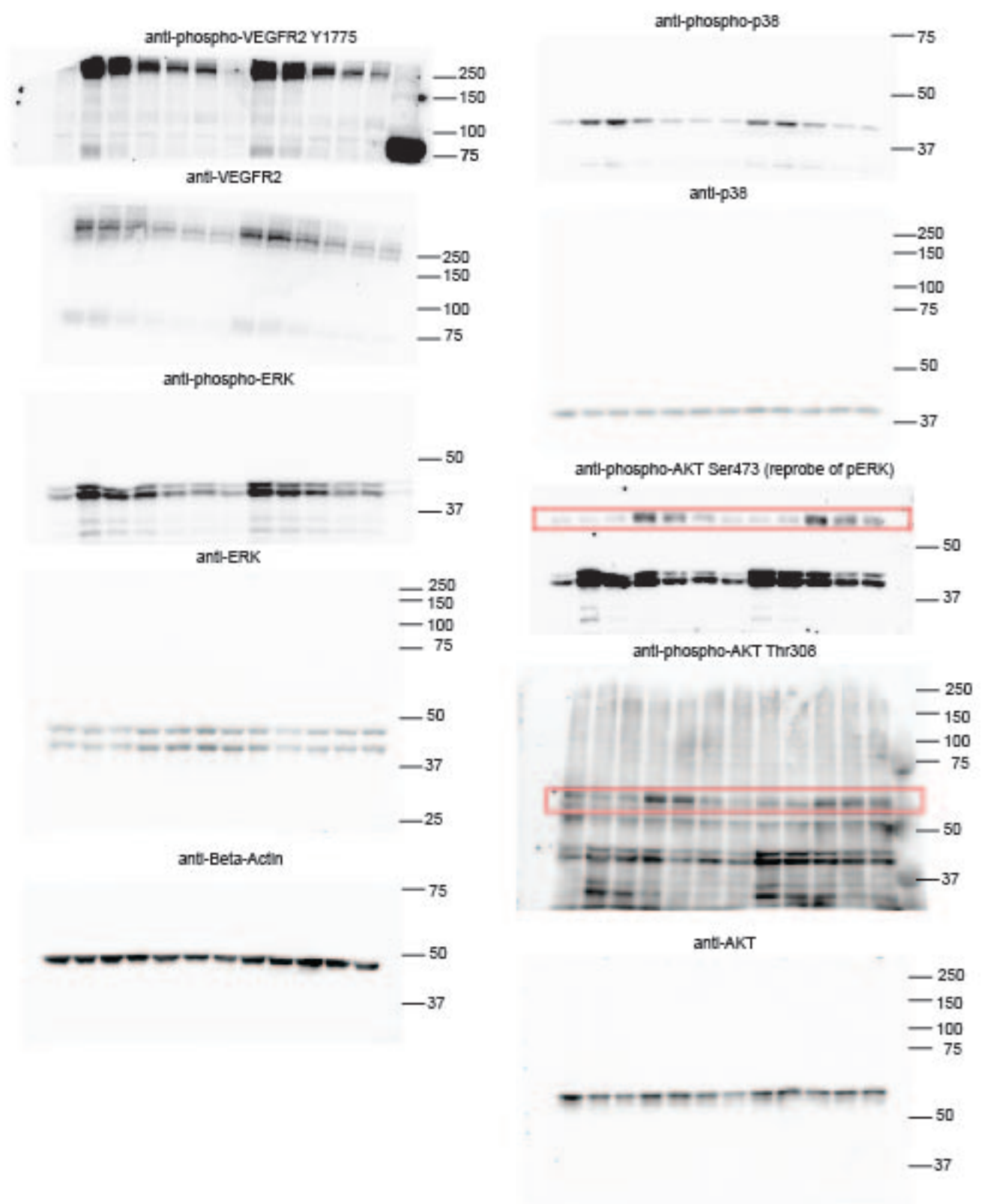

Western Blots Figure 6A

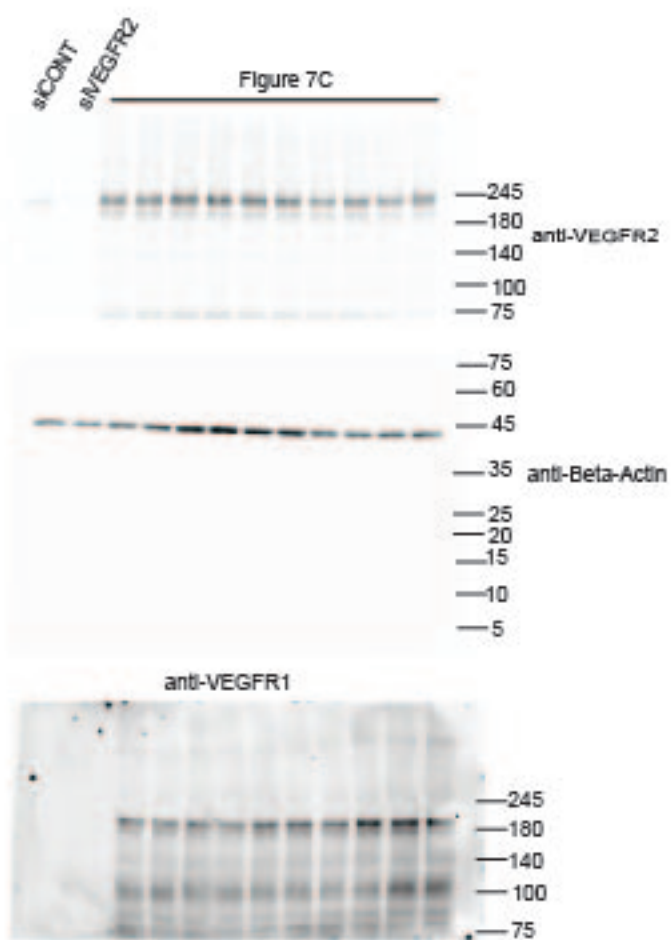

Western Blots Figure 6B

Supplement: Supplementary file 4 — Source Data for Figure 6 [file EMMM-13-e12005-s003.pdf]
